# Host-Derived Artificial MicroRNA as an Alternative Method to Improve Soybean Resistance to Soybean Cyst Nematode

**DOI:** 10.3390/genes7120122

**Published:** 2016-12-08

**Authors:** Bin Tian, Jiarui Li, Thomas R. Oakley, Timothy C. Todd, Harold N. Trick

**Affiliations:** 1 Department of Plant Pathology, Kansas State University, Manhattan, KS 66506, USA; btian@ksu.edu (B.T.); ljrluck@gmail.com (J.L.); tom@ksu.edu (T.R.O); nema@ksu.edu (T.C.T); 2 Bayer CropScience, 3500 Paramount Pkwy, Morrisville, NC 27560, USA

**Keywords:** artificial microRNA, soybean cyst nematode, soybean, resistance

## Abstract

The soybean cyst nematode (SCN), *Heterodera glycines*, is one of the most important pests limiting soybean production worldwide. Novel approaches to managing this pest have focused on gene silencing of target nematode sequences using RNA interference (RNAi). With the discovery of endogenous microRNAs as a mode of gene regulation in plants, artificial microRNA (amiRNA) methods have become an alternative method for gene silencing, with the advantage that they can lead to more specific silencing of target genes than traditional RNAi vectors. To explore the application of amiRNAs for improving soybean resistance to SCN, three nematode genes (designated as *J15*, *J20*, and *J23*) were targeted using amiRNA vectors. The transgenic soybean hairy roots, transformed independently with these three amiRNA vectors, showed significant reductions in SCN population densities in bioassays. Expression of the targeted genes within SCN eggs were downregulated in populations feeding on transgenic hairy roots. Our results provide evidence that host-derived amiRNA methods have great potential to improve soybean resistance to SCN. This approach should also limit undesirable phenotypes associated with off-target effects, which is an important consideration for commercialization of transgenic crops.

## 1. Introduction

The soybean cyst nematode (SCN), *Heterodera glycines*, is one of the most economically devastating pathogens on soybean. It is estimated that this disease causes more than one billion dollars in yield losses annually in the United States alone [1]. Current management strategies for *H. glycines* suffer from several limitations. For example, chemical nematicides often exhibit nontarget effects and increased production costs, while the effectiveness of crop rotation is limited by the dormancy of *H. glycines* eggs within the cysts, which can span many years [2]. Resistant cultivars have historically represented the best option for SCN management, but overreliance on a single resistance source (PI 88788) in cultivar development in the United States has led to concerns about intensive selection for virulence in nematode populations [3,4]. Resistant cultivars also display variable effectiveness across diverse *H. glycines* populations [4]. Thus, alternative and novel methods to control this widespread pest are needed to supplement current management strategies.

RNA interference (RNAi) is an endogenous post-transcriptional gene silencing (PTGS) mechanism conserved in eukaryotes [5,6]. In plants, two major classes of silencing RNAs, short interfering RNAs (siRNAs) and microRNAs (miRNAs), have both been successfully applied in crop engineering to improve plant production and quality by silencing specific plant endogenous genes [7,8]. Traditional RNAi knockdown is based on a hairpin construct with inverted fragments of the target gene separated by an intron. Double-stranded RNAs (dsRNAs) are processed by DICER-like (DCL) enzymes in the RNAi regulation pathway to produce small RNAs (sRNAs). RNA-induced silencing complexes (RISCs) are formed by incorporated these sRNAs into Argonaute (AGO) proteins. These RISCs recognize complementary sequences of corresponding genes and induce specific gene silencing by degrading mRNAs or inhibiting translation [9]. Similar strategies based on the specific blocking of parasitism genes by host-derived dsRNAs have also been reported to show promising effects on controlling various pathogens such as viruses, fungi, and insects [10,11,12,13].

The miRNA pathway is another gene silencing mechanism that mainly modulates endogenous gene expression by either suppression of protein translation and/or RNA cleavage. MicroRNAs are encoded by endogenous *MIR* genes. In plants, miRNAs are derived from single-stranded RNA transcripts to produce primary miRNAs (pri-miRNA), which have the ability to form an imperfect double-stranded fold-back structure. The pri-miRNAs are processed by DCL1, producing, in succession, a hairpin precursor miRNA (pre-miRNA) and a mature miRNA duplex (miRNA/miRNA*), finally resulting in a mature RNA of approximately 21 nucleotides in length that is incorporated into AGO1 to form a RISC [14]. Recently, an advantageous PTGS method—the artificial miRNA (amiRNA)-based approach—was developed to suppress plant endogenous genes in a very specific manner. The amiRNAs imitate the natural miRNA pathway to suppress targeted genes by expressing designed miRNAs using a host pre-miRNA backbone [15]. Given the fact that amiRNAs are a population of a single type of small RNAs with highly selected short nucleic acid sequences, off-target effects can be minimized in the design of the amiRNAs. It has been successfully applied as a useful tool in many plant systems to select mutants and study gene functions [16,17,18]. This strategy also produced promising results in plant antiviral resistance. The amiRNA based approach for antiviral resistance was first demonstrated by targeting virus silencing suppressor P69 and HC-Pro of turnip yellow mosaic virus and turnip mosaic virus, respectively, in *Arabidopsis* [19]. Additional examples of antiviral resistance using the amiRNA-based approach to target different viral genes including 2b gene, 3’ untranslated region (UTR), coat protein (CP), nuclear inclusion protein a and b, and silencing suppressors have been documented for cucumber mosaic virus (CMV), potato virus X (PVX), and potato virus Y (PVY) [20,21,22,23]. More recently, improved plant resistance was achieved by targeting the nucleoprotein (N) but not silencing suppressor genes (NSs) in tomato spotted wilt virus (TSWV) using a shortened miR159a precursor backbone [24]. However, evidence for improved plant resistance via amiRNA targeting other parasites is limited. The only study to date involves enhanced resistance to *Myzus persicae* in transgenic plants expressing amiRNAs targeting aphid acetylcholinesterase 2 coding gene (*MpAche2*) compared to transgenic plants with MpAChE2-specific hairpin RNA (hpRNA) [25]. To our best knowledge, the use of host-derived amiRNA to suppress nematodes parasites has not been reported for any plant system.

In the present study, three SCN fitness genes designated as *J15*, *J20*, and *J23*—which were previously determined to be associated with suppression of SCN reproduction in RNAi experiments (unpublished data)—were tested as host-derived amiRNA targets for improving soybean resistance to SCN. With confirmation of expression of these amiRNA constructs in soybean hairy roots, transgenic chimeric plants used in SCN bioassays showed significant suppression on SCN cyst and egg populations compared to control plants. This study is the first report that parasitic nematode populations can be suppressed by host-derived amiRNAs, and represents an alternative tool for improving host plant resistance to pathogens.

## 2. Materials and Methods

### 2.1. Plasmids, Vector Design, and Construction

**Figure 1:**
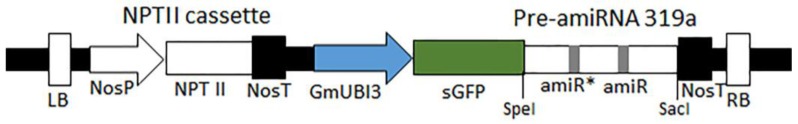
Vector map of pUFamiR construct used for host-derived gene silencing. The artificial microRNA (amiRNA) expression constructs were modified from pBI121 binary vector. The T-DNA region consists of NPTII cassette for kanamycin selection, soybean ubiquitin promoter (Gmubi3), superfolder green fluorescent protein (sGFP) reporter gene fused with the precursor amiRNA (pre-amiRNA) sequences, and the nopaline synthase (NOS) terminator at the 3’ end. The specific amiRNA for each target gene was generated by exchanging the sequences of the amiR guide and passenger (amiR*) strands within the pre-amiRNA319a gene.

To construct the pUFamiR vector for amiRNA-mediated gene silencing in legume roots (Figure 1), a 918 bp fragment of the soybean polyubiquitin (GenBank: EU310508.1) promoter (GmUbi) [26] flanked by *Not*I and *Bam*HI restriction sites was amplified via PCR from soybean genomic DNA (cultivar “Fayette”) and cloned into a modified pBI121 construct. The superfolder green fluorescent protein (sGFP) gene from HBT–sGFP–NOS construct (kind gift from Jen Sheen, Massachusetts General Hospital) was mutagenized via PCR to remove its stop codon and to flank the gene with *Bam*HI and *Xba*I sites. The gene was then cloned into the pBI121 vector to yield the pUF construct. The pre-amiRNA containing miR319a of *Arabidopsis thaliana* was cloned from pRS300, which was a gift from Detlef Weigel (Addgene plasmid # 22846) [27,28].

The 21 nucleotide (nt) artificial microRNA sequences for sGFP and three SCN genes were designed using the web miRNA designer WMD3 (http://wmd3.weigelworld.org/cgi-bin/webapp.cgi). Sequences were compared against their target sequences as well as the soybean genome *Glycine max* v109 (Phytozome). The sequences that had no or limited off-targets were used for the construction of the final pUFamiR vector (Figure 1). The miR319a backbone presented the template for the overlapping PCR to generate designed amiRNA precursor sequences for pUFamiR–gfp, pUFamiR–J15, pUFamiR–J20, and pUFamiR–J23. The construction procedure and primer design were slightly modified according to [27]. The amiRNA sequences were cloned into the pUFamiR vector at the *Spe*I/*Sac*I restriction sites. The final pUFamiR constructs were sequenced to confirm the correct amiRNA precursor sequences were as designed. All primers used in the vector construction are provided in the Supplementary Materials Table S1.

### 2.2. Hairy Root Transformation of Soybean

Soybean “KS4607” seeds, which are susceptible to *H. glycines* HG type 7 population, were transformed with *Agrobacterium rhizogenes* strain K599, and the procedure was adapted from a protocol previously described by Li et al. [29]. For each experiment, seedlings transformed with K599 containing the empty pUFamiR construct were used as controls. Three- to four-week-old composite plants growing on the selection medium were used for bioassay experiments.

### 2.3. RNA Isolation

Tissues were collected from tissue culture-derived or growth chamber-derived plants and immediately frozen in liquid nitrogen. Samples were ground with a mortar and pestle in liquid nitrogen or with a stainless-steel BB in 1.5 mL Eppendorf tubes by QIAGEN TissueLyser II (Qiagen, Valencia, CA, USA); approximately 100 mg tissue was used for each extraction, and total RNA was isolated with TRIzol (Life technologies, Grand Island, NY, USA) according to the manufacturer’s instructions.

Total RNA was isolated from collected nematode eggs feeding on transgenic roots with three biological replications in each independent experiment. Each sample was collected at the bottom of 1.5 mL tubes, and washed with sterile water at least three times. The samples were flash frozen and stored in −80 °C until used. Approximately 400 µL TRIzol reagent was added into each sample followed by three to six freeze/thaw cycles (−80 °C/37 °C) with grinding. An additional 200 µL TRIzol was added to the samples, and the solution was transferred to 2 mL Phase Lock Gel Heavy (5PRIME GmbH, Hilden, Germany), and the TRIzol protocol was continued until the clear upper phase was separated. The clear phase was mixed well with 70% ethanol, then Qiagen RNeasy Universal Mini kit was used according to the manufacturer’s instructions (Qiagen).

### 2.4. RT-qPCR Analysis

Total RNA was treated with RQ1 DNase (Promega, Madison, WI, USA) to remove genomic DNA (gDNA) contaminants. One microgram of treated total RNA was used as template in the Reverse Transcript System (Promega). The RT-qPCRs were performed in triplicate using the PowerUp^TM^ SYBR Green PCR Master Mix (Applied Biosystems, Foster City, CA, USA) on 7900HT Fast Real-Time PCR system (Applied Biosystems) with the following parameters: 95 °C for 3 min; 40 cycles (95 °C for 10 s; 60 °C for 1 min); 95 °C for 10 s, and a melt curve analysis from 60 °C to 95 °C at a ramp rate of 0.1 °C/s. The melt curve analysis was used to confirm the specificity, and RT-qPCR amplicons were sequenced to confirm that the target gene was amplified. The soybean GmACTII and GmEF1b [30] were both used as internal controls to normalize expression of each target gene in plant, and the *H. glycines* β-actin (AF318603) was used as the internal control in nematodes. The primers of stem-loop PCR were designed according to Chen et al. [31] with some modifications [32]. Finally, the comparison of relative gene expression levels was calculated by 2^−ΔΔ*C*t^ method [33]. Three biological replications, each consisting of a pooled sample from 4 of the 12 positive transgenic roots, as well as three technical replicates, were used for each construct in each independent experiment. A list of primers used in this study can be found in Supplementary Materials Table S1.

### 2.5. SCN Bioassay

To investigate the capacity of transgenic plants to suppress SCN reproduction, we generated composite soybean plants used for SCN bioassays. Positive composite plants expressing pUFamiR constructs were transplanted from 4-week-old tissue culture into D40 Deepots (Stuewe and Sons, Inc., Corvallis, OR, USA) with naturally infected soil containing approximately 3600–4000 eggs per 100 cm^3^ of an HG type 7 (Race 3) *H. glycines* population in each independent assay. The SCN population originated from a naturally infected soybean field in Cherokee Co., KS, and was maintained in the greenhouse on susceptible soybean cultivar KS3406. The susceptible soybean cultivar KS4607 inoculated with K599 containing a pUFamiR empty vector was used as the negative control. The composite plants were grown in a growth chamber under 26 °C/24 °C and 16/8 h light/dark photoperiod, with the experimental treatments arranged in a completed randomized design. After 5 weeks of plant growth, the number of SCN cysts and eggs from individual plants were counted. Independent experiments, each consisting of an empty vector control and at least two of the three constructs were conducted in an augmented design. Each construct was represented by at least 12 positive transgenic roots for each experiment, and was present in at least three independent experiments.

Interactions between experiment and treatment were not observed in preliminary analyses of variance, indicating that these effects were additive. Based on these results, the effect of transgenic construct on SCN cyst and egg numbers was analyzed using the GLMMIX procedure in SAS (SAS Institute, Cary, NC, USA), with experiment treated as a random effect, and the negative binomial distribution used to account for heterogeneity of variances. Least squares means (LSMEANS) were employed to compare numbers of SCN cysts and eggs on transgenic versus control plants.

### 2.6. GFP Imaging

After selection on ½ strength Murashige and Skoog (MS) + 200 µg/µL kanamycin medium, root tips were imaged using a Leica microscope with a GFP filter cube and Kodak Digital Image Acquire software 3.0 (Kodak, Rochester, NY, USA). Blue-light images were taken with different exposure times for better results.

## 3. Results and Discussion

### 3.1. Identification of miRNA Targets

Three *H. glycines* genes encoding proteins with essential functions, designated as *J15*, *J20*, and *J23*, were selected as the amiRNA targets for this study based on RNAi phenotype data derived from *Caenorhabditis elegans* available through WormBase (http://www.wormbase.org/). The gene *J15* (Nematode.net accession HG02060) encodes an actin-related protein that has 75% similarity with *C. elegans* actin-related protein 3 (*arx-1*) (NM_058665.4). *arx*-*1* is an essential gene of *C. elegans*, as its inhibition results in embryogenesis disruption and reduced egg production [34]. The gene *J20* (Nematode.net accession Hg01053) may encode a phosphatase precursor orthologous to *C. elegans unc-26* gene. Phosphatases usually play an important role in signaling pathways, influencing numerous cellular responses, including calcium ion mobilization, protein phosphorylation, and cell proliferation [35,36]. The gene *J23* (Nematode.net accession HG03370) encodes a putative actin-binding protein, and also shared over 70% similarity with *C. elegans* Profilin-1 (*pfn*-1), a gene that affects the structure assembly of the cytoskeleton [37].

### 3.2. Construction of an amiRNA Gene Silencing Vector

**Figure 2:**
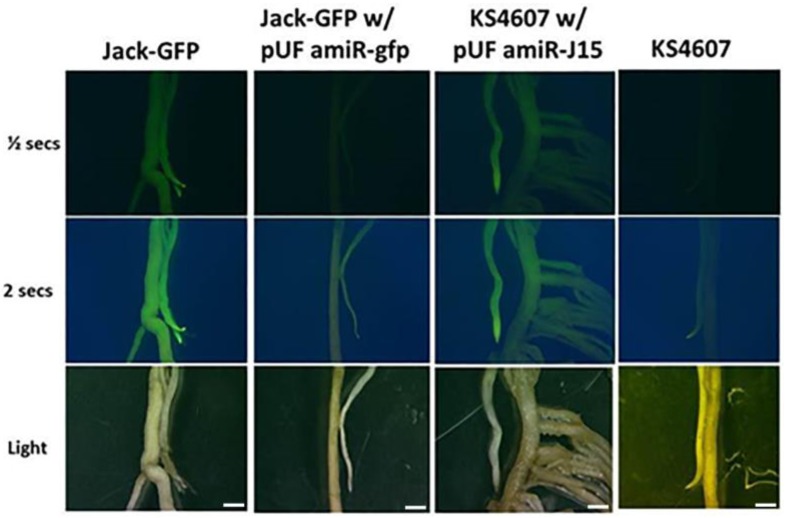
Validation of the pUFamiR vector. Row 1 and Row 2: visualization of GFP expression observed under blue light. Row 3: roots observed under white light. Column 1: roots from a transgenic soybean cultivar “Jack” expressing GFP. Column 2: Jack-GFP line transformed with pUFamiR-gfp. Column 3: cultivar KS4607 transformed with pUFamiR-J15. Column 4: nontransformed control KS4607. Scale Bar = 1 mm.

A new binary vector for gene silencing in transgenic soybean hairy roots was constructed to aid in the identification of transformed tissue (Figure 1). Driven by the soybean ubiquitin 3 promoter [38], the sGFP reporter gene was fused on the upstream sequence of pre-amiRNA to facilitate the identification of transgenic roots by GFP screening, because of the heterogeneous differentiation of the composite plant roots. The GFP expression *in planta* was confirmed by Agrobacterium inoculation with pUFamiR-J15 constructs on KS4607 cultivar roots, and the fluorescence intensity was comparable to the stable transgenic GFP plant (Figure 2, first and third column).

The transgenic chimeric plants were likely to generate nontransgenic roots when they were continuously grown without antibiotic selection. This could result in unexpected cyst nematodes grown on nontransgenic roots of transgenic chimeric plants. The GFP visible marker could facilitate monitoring and selecting of the positive hairy roots, whereas fused GFP may affect the expression levels of amiRNAs *in planta* [39,40]. In a future study, stable transgenic plants with a constitutive strong promoter driving amiRNA constructs need to develop for improving gene-silencing efficiency for targeted SCN genes.

### 3.3. Confirmation of the Functioinality of the pUFamiR Vector

**Figure 3:**
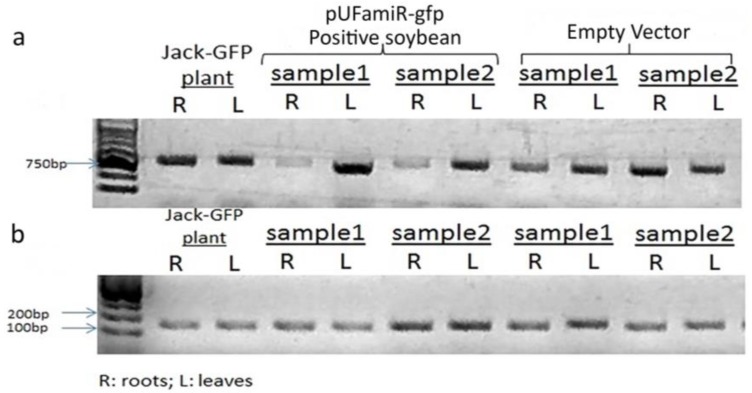
RT-PCR analysis of typical transgenic root samples to confirm the expression level of sGFP mRNAs. (**a**) The GFP expression was tested on the root tissue and leaf tissue separated with the positive and control transgenic hairy roots, independently. Control soybean was inoculated by K599 with pUF empty vector. The Jack–GFP transgenic plants were used as a positive control; (**b**) The expression level of the *Rib* gene was monitored in parallel from the same samples as a control.

The ability of the pUFamiR vector to induce silencing was confirmed by targeting the sGFP gene in a constitutively expressed soybean event previously engineered [38]. The loss of GFP expression in plant roots after transformation with the pUFamiR–gfp construct was observed as expected (Figure 2, second column). The abundance of sGFP mRNA was significantly reduced in root tissues as shown in RT-PCR (Figure 3). On the other hand, the amiR–gfp produced in the hairy roots did not appear to migrate to nontransformed tissue of the seedling. The sGFP mRNA levels in leaves showed no difference between pUFamiR–gfp-inoculated plants and controls. The fusion sGFP protein expressed by the pUFamiR vector was functional as demonstrated by GFP fluorescence on hairy roots of the nontransgenic cultivar, KS4607. Using the pUFamiR–J15 construct, GFP fluorescence was observed within the transformed hairy roots due to the GFP reporter in the construct (Figure 2, third column).

The GFP expression and silencing experiments confirmed that the pUFamiR vector containing the fused GFP reporter could express in the plants, and the pUFamiR-gfp vector that was targeting GFP in the transgenic plant could induce designed gene silencing efficiently, as expected. The pre-miRNA fused with sGFP did not affect the mRNA expression and miRNA production in the plant, and it appeared to be expressed as an endogenous pathway in the plant [7,40].

### 3.4. Expression of amiR Constructs Targeting Nematode Genes in Soybean Hairy Roots

**Figure 4:**
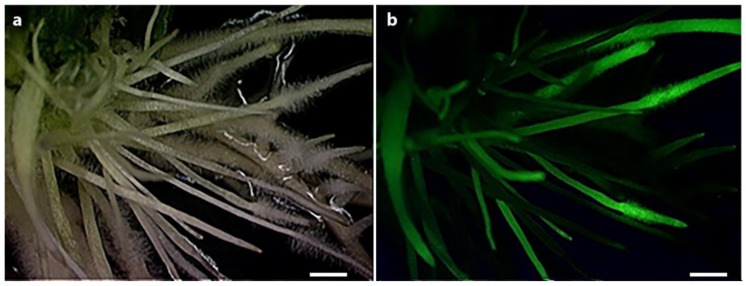
The typical expression of soybean hairy roots transformed with pUFamiR constructs containing GFP reporter. The observation of transgenic roots after 3 weeks grown on selection medium. (**a**) The positive root structure was observed under bright field; (**b**) The positive root structure was observed under blue light. Scale bar stands for 1 mm.

To select expressed constructs in hairy roots, a fluorescent sGFP gene was cloned on the upstream region of pre-amiRNA sequences as visible marker for selection (Figure 1). As shown in Figure 4, the transgenic growing roots in the selection medium presented strong fluorescence under the blue light, indicating the constructs were successfully expressed. It is always expected that a portion of roots generated from chimeric plants do not carry the designed gene constructs [41].

**Figure 5:**
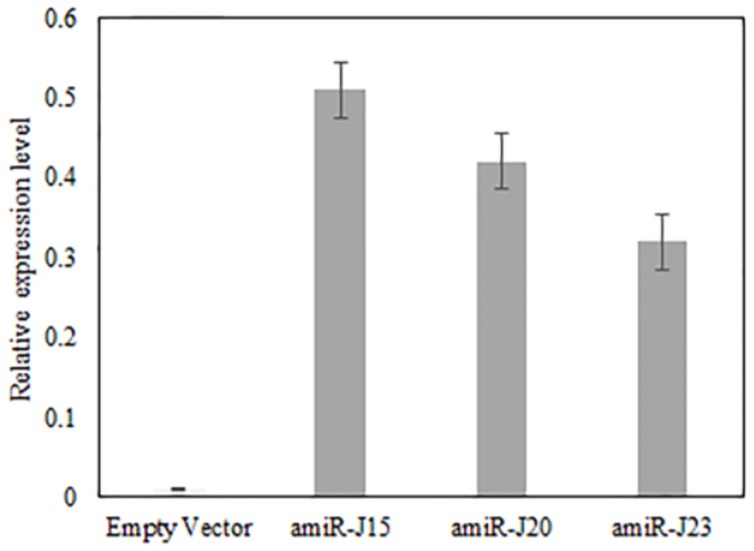
The stem-loop RT-qPCR confirmed the expression of amiR-J15, amiR-J20, and amiR-23 in the hairy roots but not in the hairy roots with empty vector. All three amiRNAs were not detected in the empty vector. Error bars represent the standard error of the mean for three experiments and three biological replicates per experiment, with experiment treated as a random effect.

To confirm the expression and accumulation of designed amiRNAs, the stem-loop RT-qPCR was performed for all positive transgenic hairy roots. It has been shown to provide better specificity and sensitivity for small RNA quantitation [31,32]. The results (Figure 5) indicated the mature miRNAs had significantly accumulated in the transgenic hairy roots. The amiR-J15 had relatively high expression compared to the other two constructs in the soybean hairy roots.

### 3.5. Bioassay of SCN Production on Transgenic Hairy Roots Expressing pUFamiR Constructs Targeting Nematodes Genes

**Figure 6:**
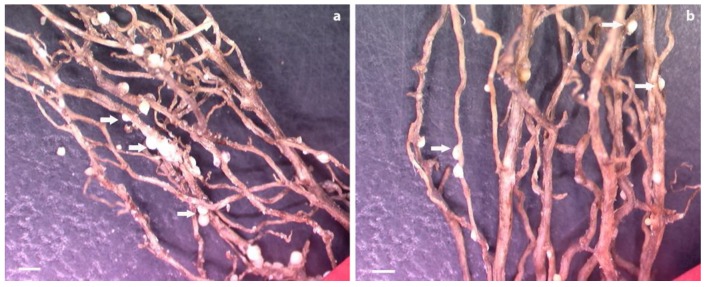
Roots from controls and composite plants infected with soybean cyst nematode (SCN) at 5 weeks postinoculation. (**a**) Roots transformed with pUFamiR empty construct showing numerous cysts (arrows); (**b**) roots transformed with pUFamiR–J15 showing significantly less cyst density. Scale b3ar = 1 mm.

**Figure 7:**
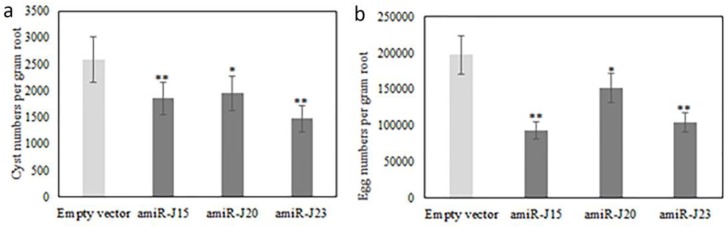
Comparison of *H. glycines* cyst (**a**) and egg (**b**) densities on roots of transgenic composite plants. Composite plants transformed with the empty vector were used as negative controls. Bars with one or two asterisks are significantly different from the empty vector control at *p* < 0.05 (*) and 0.01 (**), respectively. Error bars represent the standard error of the mean based on at least three independent bioassay experiments, with experiment treated as a random effect.

Bioassays were conducted by transplanting composite plants independently transformed with pUFamiR-J15, pUFamiR-J20, and pUFamiR-J23 into soil uniformly infected with *H. glycines* to detect whether any suppression of *H. glycines* development and/or reproduction occurred on transgenic hairy roots. All transformations of the composite plants were verified by PCR analysis. Visual inspection of the roots at the completion of the bioassays revealed differences between negative controls and transgenic roots (Figure 6). Control plants with the empty pUFamiR vector had mean values 2582 cysts per gram root (493 cysts per plant), and 197,521 eggs per gram root (36,525 eggs per plant). Reductions of 28% and 43% in numbers of cysts per gram root were observed for amiR-J15 lines and amiR-J23 lines, respectively, compared with control plants (*p* < 0.01) (Figure 7a). The amiR-J20 plants reduced cyst densities by 24%, however, there was less evidence of an effect for this construct (*p* = 0.017). Similar results were obtained for egg densities, with amiR-J20 plants showing the smallest reduction of 23% (*p* = 0.0204), while the amiR-J15 and amiR-J23 lines produced reductions of 53% and 47% (*p* < 0.01), respectively (Figure 7b).

Composite soybean plants generated from each pUFamiR construct with different targeted genes were examined in three independent bioassay experiments to produce robust and reliable data. All of the tested lines exhibited significant reductions in both cyst and egg numbers as compared with controls. The amiR-J20 lines showed the least reduction in both cyst and egg densities, possibly due to a less critical function of the selected gene in SCN parasitism, and/or the presence of similar homologous functional genes or related gene families that could complement the lost function of *J20*, as the targeting of an amiRNA construct was likely very specific based on the short sequence [7].

For amiR-J15 lines, the average reduction in cyst numbers densities was relatively low (28%), whereas this line produced the largest reduction in egg density (53%). A preliminary hypothesis is that this actin-related gene was likely more essential for SCN reproduction than for the infection and feeding site establishment process. The amiR-J23 lines showed similar reductions for both cyst and egg densities.

Nematode bioassays on transgenic hairy roots could always generate variable results depending on the transformation and expression levels of each construct in different events, as well as the condition of nematode culture [42,43]. The mixed transgenic hairy root (as shown in Figure 4) could also affect the SCN survival rate. Composite plants were grown in the soil for five weeks during the bioassay, and some nontransgenic root growth were unavoidable. The presence of root mixtures could inflate cyst and/or egg numbers on transgenic roots, making our observed effects conservative. Stable transgenic lines need to be developed for each construct to confirm the conclusions from this study. Nonetheless, the hairy root system is still a suitable approach for exploring parasitism and development genes in nematodes, as well as for selecting effective genes for engineering crop plant cultivars for nematode management [15]. A number of reports have used the hairy root system to evaluate the interaction among the plant host and nematodes [29,44,45]. With ever-increasing genome-sequencing data, many additional genes essential for parasitism, development, and reproduction are likely to be discovered, facilitating the process of effectively engineering crop plant resistance to SCN and other nematode pests.

### 3.6. Downregulation of Candidate Genes in Nematodes Feeding on Composite Hairy Roots Expressing the pUFamiR Constructs

**Figure 8:**
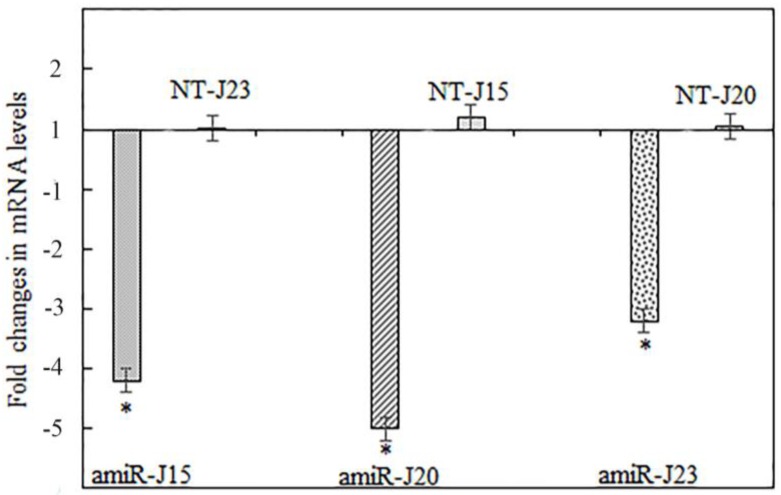
Real-time RT-qPCR results showing the downregulation of target *H. glycines* genes *J15*, *J20*, and *J23* at mRNA transcript levels by host-derived amiRNA determined at 5 weeks after inoculation. The *H. glycines* β-actin gene was used as an internal control to normalize gene expression levels among samples. Nematodes feeding on composite plants with the pUFamiR empty vector were used as control samples. The 2^−ΔΔ*C*t^ method was used to quantify the relative change in gene expression. The relative expression of a nontarget (NT) parasitism gene in each experiment remained unchanged. Error bars represent the standard error mean between three independent bioassay replicates. * *p* < 0.05.

It is important to determine whether the delivery of small RNAs and/or miRNAs through oral uptake by SCN leads to downregulation of nematode parasitism gene expression corresponding with reduced fitness on host plants. The observed effect on nematodes establishment and reproduction depends on the importance of the gene targeted and the level of mRNA suppression [46]. To determine whether the targeted genes in SCN were downregulated by expressed pUFamiR constructs in present study, total RNAs extracted from nematodes feeding on roots of soybean hairy roots at five weeks post inoculation were analyzed for the transcript abundance in each sample by RT-qPCR. The results indicated that all three targeted *H. glycines* genes had downregulated mRNA transcripts in nematodes collected from transgenic plant roots with the corresponding pUFamiR constructs. The observed downregulation of targeted genes was a 3.2- to 5-fold reduction compared to controls that were inoculated with pUFamiR empty vector only (Figure 8). The decrease in the transcript levels of all three parasitism genes was statistically significant (*p* < 0.05), whereas the transcript levels of nontarget parasitism genes were not statistically changed (Figure 8). As expected, all tested parasitism genes illustrated specific mRNA downregulation in SCN eggs recovered from transgenic roots with corresponding pUFamiR constructs.

The nematodes feeding on amiR-J20 plants showed the largest reduction of the targeted *J20* gene, however, this reduction did not correspond to the highest reduction in nematode population density of the two traits tested in the bioassay. Lack of correlation is possibly due to the complementary functions of other homologous genes in the nematodes [15]. It has been reported that the efficiency of amiRNA-based gene silencing can be affected by various factors in plants, including the gene and design, the expression level, accessibility of the complementary site on target mRNA, and the negative feedback regulation of target RNA level [46]. In addition, the uptake of molecules through nematode stylets and feeding tubes is known to be selective and variable among nematode genera [47]. The mechanism and process of miRNA molecules being delivered into cyst nematodes and then triggering silencing pathways are still not clear. It is plausible that amiRNA constructs targeting different regions of the same gene may significantly impact the levels of gene silencing [48].

Currently, there is a growing body of evidence that plant-derived siRNA can provide nematode resistance [15,29,42,49,50,51,52]. Large reductions (81%–93%) in *H. glycines* cysts were obtained from transgenic roots by silencing either two ribosomal proteins, a spliceosomal protein or synaptobrevin using the hpRNAi approach [53]. Similar reductions in SCN egg production using soybean transgenic hairy roots were also achieved by hpRNAi targeting *Prp-17* and *Cpn-1* [42]. The present study demonstrates that amiRNA produced *in planta* can have similar effects on soybean cyst nematodes and suggests that the SCN feeding tube allows miRNA delivery from host plants.

Large variations in nematode reduction using a host derived hpRNAi based approach was commonly observed among and within the transgenic RNAi lines [50,52,54,55]. These variations could potentially be explained by the fact that the delivery mechanisms of small RNA molecules between plants and nematodes and its limitations are unknown [56], transcriptional and/or post-transcriptional gene silencing of the transgenes may occur [57], and the individual parasitism and/or development genes can also play different roles during the infection. Although large variations were not observed in this study, additional studies using host-derived amiRNA are needed. As amiRNA constructs have relatively short sequences and allow vector design flexibility, a single amiRNA construct stacking with several parasitism, development, and/or production gene targets could likely boost the soybean resistance level for SCN in the further studies [40].

## 4. Conclusions

The host-derived amiR-mediated gene silencing system presented here provides important evidence that miRNAs can be delivered to plant parasites, such as nematodes, triggering the endogenous gene silencing in the pathogen. This could be an alternative tool for managing soil-borne pathogens with lower risks of off-target effects than the traditional hpRNAi silencing strategy. In addition, individual amiR-silencing of two SCN genes, *J15* and *J23*, separately produced moderate levels of SCN resistance in transgenic soybean roots. This method could be applied to screening other potential target genes in nematodes. Further, this amiRNA-based biotechnology could be regarded as an alternative approach for intragenic crop engineering that may cause less concern in the public [18]. We are confident that this strategy will lead to more specific control of other agriculturally important pests and pathogens in the near future.

## Supplementary Materials

The following are available online at http://www.mdpi.com/2073-4425/7/12/122/s1, Table S1: Primer sequences used in the present study.
